# Molecular characterization of a new highly divergent Mobala related arenavirus isolated from *Praomys* sp. rodents

**DOI:** 10.1038/s41598-021-88046-5

**Published:** 2021-05-13

**Authors:** Huguette Simo Tchetgna, Stephane Descorps-Declère, Benjamin Selekon, Aurelia Kwasiborski, Mathias Vandenbogaert, Jean-Claude Manuguerra, Antoine Gessain, Valérie Caro, Emmanuel Nakouné, Nicolas Berthet

**Affiliations:** 1 Centre for Research in Infectious Diseases, Yaoundé, Cameroon; 2 The Center for Microbes, Development and Health, CAS Key Laboratory of Molecular Virology and Immunology, Institut Pasteur of Shanghai – Chinese Academy of Sciences, Discovery and Molecular Characterization of Pathogens, Shanghai, 200031 China; 3 Center of Bioinformatics, Biostatistics and Integrative Biology (C3BI), Institut Pasteur, Paris, France; 4 Institut Pasteur de Bangui, Bangui, Central African Republic; 5 Cellule d’Intervention Biologique d’Urgence, Institut Pasteur, Unité Environnement et Risques Infectieux, Paris, France; 6 Unité d’Epidémiologie et Physiopathologie des Virus Oncogènes, Département de Virologie, Institut Pasteur, Paris, France; 7 Centre National de Recherche Scientifique (CNRS) UMR3569, Paris, France

**Keywords:** Bioinformatics, Sequencing, Virology, Arenaviruses

## Abstract

Arenaviruses represent a family of viruses that are naturally present in rodents belonging to subfamily Murinae, Neotominae or Sigmodontinae. Except for Lassa virus, little information is available on other Old-World arenaviruses. Here, we describe strain AnRB3214, a virus isolated from a presumed *Praomys* sp. rodent in the Central African Republic in 1981 and assigned to Ippy virus based on antigenic similarity. The strain was simultaneously sequenced on Illumina NovaSeq 6000 and MinION Mk1B devices and analysed with various bioinformatics tools. We show that the best genome coverage and depth were obtained with the Kaiju and Minimap2 classification and identification tools, on either the MinION or the Illumina reads. The genetic analysis of AnRB3214 fragments showed 68% to 79% similarity with the Mobala and Gairo mammarenaviruses at the nucleic acid level. Strain AnRB3214 had a truncated nucleoprotein smaller than that of other Old World arenaviruses. Molecular clock analysis suggests that this strain diverged from Mobala virus at least 400 years ago. Finally, this study illustrates the importance of genomics in the identification of archived viruses and expands on the diversity of African arenaviruses, because strain AnRB3214 is either a variant or a close relative of Mobala virus, and not Ippy virus.

## Background

Arenaviruses are enveloped, single-stranded RNA viruses belonging to family *Arenaviridae*, order *Bunyavirales*. With approximately 43 species, arenaviruses are subdivided into four genera (*Antennavirus*, *Hartmanivirus*, *Reptarenavirus* and *Mammarenavirus*) that infect fishes, snakes or mammals^1^. Arenaviruses typically have ambisense, bisegmented RNA genomes. Each RNA segment encodes two non-overlapping open reading frames (ORFs) of opposite polarity. The small (S) 3500 nt genomic segment encodes the nucleocapsid protein (NP) and a glycoprotein precursor (GPC), which later matures into two membrane glycoproteins, GP1 and GP2. The large (L) ~ 7000 nt genomic segment codes for the RNA-dependent RNA polymerase (RdRp) and exceptionally a zinc-binding protein (Z) in the *Mammarenavirus* and *Reptarenaviru*s genera^2,3^. Interestingly, *Antennavirus* has a trisegmented RNA genome, with NP and GPC being on two distinct segments^4^.

Mammarenaviruses are geographically segregated into two monophyletic groups based on genomic features and antigenic properties. The New World (NW) arenaviruses, represented by the Tacaribe virus complex, are prevalent in North and Latin America; the Old World (OW) arenaviruses, represented by the Lassa-Lymphocytic choriomeningitis virus (LCMV) complex, are found in Africa^3,5^. The natural reservoirs of mammarenaviruses are generally rodent subfamilies *Murinae*, *Neotominae* and *Sigmodontinae*, in which they establish persistent infections with no overt pathology for the animal host^6,7^. These viruses seem to be associated with specific rodent host species, although many host-switching events have been recorded, which implies that virus-host co-evolution may not be as tightly linked as previously thought^6,8,9^. Most mammalian arenaviruses are non-pathogenic to humans, but seven are recognized as haemorrhagic fever viruses including the Lassa and Lujo viruses in Africa and the Sabia, Machupo, Chapare, Junin and Guaranito viruses in Latin America^10,11^. LCMV, which has a worldwide distribution, can cause neurological disease in immune-compromised individuals^12^. Except for Lassa virus, knowledge of OW arenavirus diversity, evolution and involvement in human diseases is sparse. Several arenaviruses of unknown pathogenic potential have been described in African rodents such as Gbagroube, Kodoko and Menekre viruses in West Africa and Mopeia, Morogoro, Luna, Merino Walk, Mariental and Okahandja viruses in southern Africa^2^. In Central Africa, wild and domestic rodents act as reservoirs of numerous parechoviruses, rhabdoviruses, phleboviruses, flaviviruses, picornaviruses, astroviruses, paramyxoviruses, orthopoxviruses and arenaviruses, some of which are directly responsible for human diseases, including monkeypox, Mokola virus infection or lymphocytic choriomenigitis^13–15^.

Few arenaviruses are known in Central African rodents; however, LCMV, Souris, Mobala and Ippy viruses have been described in *Mus*, *Praomys*, *Mastomys* and *Arvicanthis* rodents in Gabon, Cameroon and the Central African Republic (CAR)^13,16–18^. Seroconversion has been highlighted in the CAR for Mobala virus infection in humans in locations where the virus has been isolated from *Praomys* rodents^17,19^. Interestingly, seroprevalence in humans seems to reflect virus isolation in its reservoir host. Indeed, for a prevalence of 7.7% and 3.2% in *Praomys* sp. rodents in the Bouboui and Gomoka villages, respectively, using the indirect fluorescent antibody test (IFAT), the prevalence in humans ranges from 3.1 to 3.8%; however, in Botambi, with a prevalence of 2.2% in the reservoir, no antibodies have been detected in the human population^19^. Seroconversion in humans for Souris and Ippy viruses remains to be determined.

Next-generation sequencing (NGS) platforms have been a breakthrough in many scientific fields. In virology, NGS has facilitated a better understanding of the diversity and evolutionary history of viruses, the discovery of new viruses and the rapid response to outbreaks through diagnosis of the causative agents^4,20–22^. The leading NGS technologies, among which Illumina, are based on the incorporation of terminating nucleotides in a DNA polymerization process. These technologies, which produce very accurate and high-quality results, are widely used. However, they are usually cumbersome and only available in well-equipped, specialized laboratory facilities that can provide all the necessary utilities, such as a stable electric supply and internet connection. On the other hand, MinION uses a different approach in which a specific nanopore protein is used to recognize a k-mer in the sequence of interest via a disturbance in the basal electrical current. This innovation led to the miniaturization of the sequencing device and the ability to perform real-time sequence analysis^23,24^. This portable tool is gaining interest because it brings more flexibility to sequencing, albeit with a high error rate, and can, therefore, be used in remote regions as a diagnosis tool or in response to outbreaks as seen in the recent Lassa, Ebola and Zika epidemics^22,25–29^. Despite its shortcomings, MinION sequencers can be implemented in resource-limited settings such as the CAR as a first-line tool to respond instantly to emergencies or to analyse infectious samples subject to transportation restrictions.

Here, we describe a divergent arenavirus, the AnRB3214 strain isolated from wild *Praomys* rodents in the CAR in 1981. The strain was sequenced using the MinION Mk1B (Oxford Nanopore Technologies, Oxford, UK) and Illumina NovaSeq 6000 (Integragen, Ivry, France) devices and analysed with various bioinformatics pipelines.

## Methods

### Description of the strain

**Figure 1:**
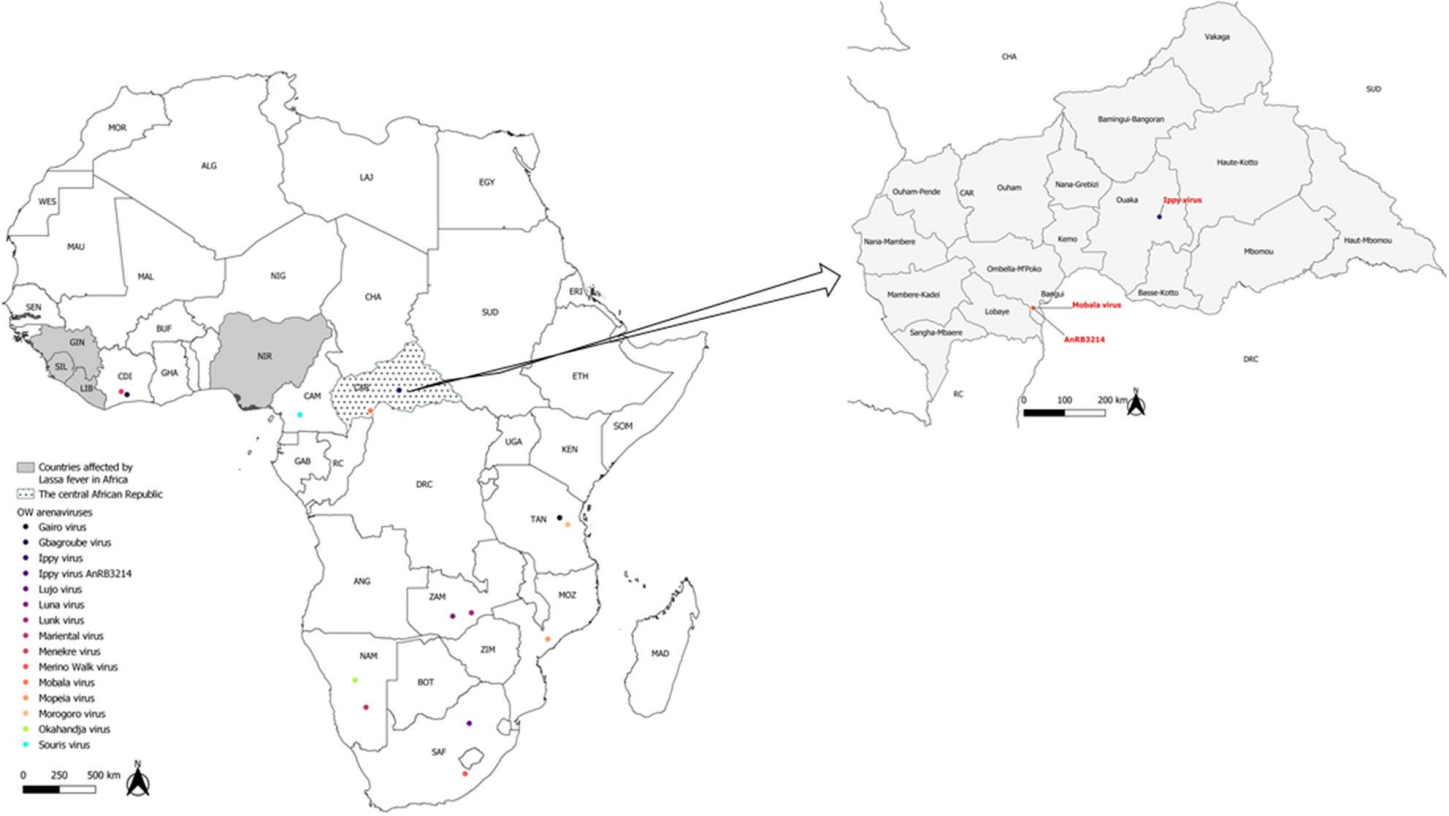
Distribution of Old word arenaviruses across Africa. Coloured circles indicate countries in which OW arenaviruses have been isolated. Countries shown in grey are those where Lassa outbreaks have been recorded in humans and the virus detected in various rodents such as *Mastomys natalensis*. The CAR is highlighted (dot-filled). Ippy virus has been identified in the Ouaka prefecture, and Mobala and AnRB3214 (formerly identified as Ippy) originate from the Ombella-M’poko prefecture. The maps were drawn in QGIS v3.14.16 (https://www.qgis.org/fr/site/) using geopackages provided by GADM v3.6 (https://gadm.org/index.html).

Strain AnRB3214, initially identified as Ippy virus, was isolated from a presumed *Praomys* sp. rodent caught near Botambi, Ombella-M’poko prefecture, CAR in October 1981 (Fig. 1). The initial isolation procedure was performed by intracerebral infection of two-day-old newborn mice up to the sixth passage. The viral isolate was then freeze-dried (lyophilized) for long-term conservation at room temperature.

### Virus isolation

A volume of 0.2 mL of 1X PBS-diluted lyophilized virus was used to inoculate a litter of eight newborn mice (24–72 h old). Only mice that died after a 7-day follow-up were harvested and their brains were collected, crushed and suspended in a 1X PBS solution, and then filtered. This filtered brain suspension was used to infect fresh confluent Vero E6 cells culture in Dulbecco’s Modified Eagle’s Medium (DMEM, Gibco, Life Technologies, Carlsbad, California, USA) supplemented with 2% Foetal Calf Serum (FCS, Gibco, Life Technologies) and a 1% v/v antimicrobial solution (10,000 units/mL of penicillin, 10,000 µg/mL of streptomycin and 25 µg/mL of amphotericin B, Gibco, Life Technologies). The cell cultures were monitored for 7 days for cytopathic effects using an inverted optical microscope. The brain suspensions and cell culture supernatant were then stored at -80 °C until further use.

### Nucleic acid extraction

Total RNA was extracted from the culture supernatant using the QIAamp Viral RNA Mini kit (Qiagen, Hilden, Germany) according to the manufacturer's protocol. These RNAs were then quantified using the Qubit RNA HS Assay kit (Invitrogen, Life Technologies, Carlsbad, California, USA) and stored at -80 °C.

### Preparation of libraries for Illumina and MinION sequencing

The first cDNA strand was prepared using SuperScript III reverse transcriptase and random hexamers (Invitrogen, Life Technologies, Carlsbad, California, USA) on total RNA. The second cDNA strand was then prepared using the NEBNext Ultra II Non-Directional RNA Second Strand Synthesis Module kit (New England BioLabs, Hitchin, Hertfordshire, UK) according to the manufacturer's instructions. All final repair and adapter ligation steps were based on the use of the NEBNext Ultra II RNA Library Prep Kit for Illumina (New England BioLabs, Hitchin, Hertfordshire, UK). All DNA purification steps were performed using Agencourt AMPure XP beads (Beckmann Coulter, Woerden, Netherlands). The library quality was checked on the 2100 BioAnalyzer instrument (Agilent Technologies, Santa Clara, California, USA) and then sequenced on Illumina NovaSeq 6000 sequencer (Integragen, Ivry, France) to obtain 2 × 150 bp paired-end reads. In parallel, the double-stranded cDNA obtained after the end repair step was used to prepare the MinION library with the PCR Barcoding kit (SQK-PBK004) (Oxford Nanopore Technologies. Oxford, Oxfordshire, UK) according to the manufacturer's instructions. The library was multiplexed with five other samples and loaded on a R9.4 flow cell (FLO-MIN106) and sequenced on the MinION Mk1B device in 37 h. The raw sequencing data were collected using ONT MinKNOW software (version 19.05.0).

### Bioinformatics analysis

The quality of the raw reads from the Illumina sequencing was first assessed, filtered, and then trimmed using CLC Genomics workbench v10.0.1 (Qiagen, Hilden, Germany). All trimmed reads were then de novo assembled using SPAdes v3.10 to obtain two contigs corresponding to the S and L segments of an arenavirus genome. All reads were mapped back on the two contigs previously obtained using Geneious Prime (Biomatters, Auckland, New Zealand). All the reads from the Illumina sequencing that have been mapped were grouped together in a constituted dataset referred to as "AnRB-3214-Illumina". Three subsets, derived from the AnRB-3214-Illumina dataset, were constituted by collecting random reads from the raw fastq files in such a way that the first dataset represented 10% of the initial reads, the second 1%, and the last 0.1%. In parallel, the raw data obtained from MinION sequencing were acquired in real-time in files containing 4000 sequences in FAST5 format. These files were base-called and demultiplexed using Guppy (version 3.4.1). As carried out for the Illumina raw reads, the MinION raw reads were mapped on the obtained sequences of the two segments using Minimap2^30^. All the reads that have been mapped were grouped in a dataset referred to as "AnRB-3214-MinION". From this dataset, three sub-datasets were constituted in the same way as previously. Viral diversity was determined from the two above-described datasets (AnRB-3214-MinION and AnRB-3214-Illumina) and their three sub-datasets using three different taxonomic classification tools: Kaiju^31^, Centrifuge^32^, and Kraken2^33^. These tools use reference databases in which the sequence of our AnRB-3214 strain was absent. In parallel with the use of these three taxonomic classification tools, two other traditional approaches were used for the identification of viral reads. The first approach consisted in a homology search from reference sequences using the BLASTN tool whereas the second approach was to map the reads on other reference sequences using Minimap2. To use these approaches, the genomes of viruses closest to our AnRB3214 strain were screened for in GenBank and identified as Mobala virus (DQ328876, AY342390) and Gairo virus (KJ855308, KJ855307). Then, we screened the AnRB-3214-MinION and AnRB-3214-Illumina datasets against these Mobala and Gairo virus sequences using the BLASTN (https://blast.ncbi.nlm.nih.gov/Blast.cgi) and Minimap2 tools. Hits produced by both methods were reformatted in fastq files and remapped to obtain bam files for both BLASTN and Minimap2. Bam files were converted into consensus sequences by applying BCFtools on the results of SAMtools mpileup.

### Phylogenetic analysis and molecular clock estimation

S and L segment nucleic-acid sequences as well as the coding sequences of the NP, GPC, RdRp and Z genes were aligned using ClustalO in Unipro Ugene version 34.0^34^ and manually edited. Then, phylogenetic trees were inferred for each segment using the maximum-likelihood (ML) method implemented in MEGA X (version 10.1.1) under the GTR + I + Γ4 nucleotide substitution model as determined by the best model finder in MEGA X^35^. The node supports were estimated using 1000 bootstrap replicates.

Concurrently, a molecular clock was tested on concatenated S and L segments using Bayesian inference. S + L concatenates of genomic sequences were aligned using MAFFT v7^36^ and manually edited with AliView^37^. Starting from this alignment, ML and Bayesian analyses were conducted. A global ML evolutionary tree was reconstructed using IQ-TREE^38^ according to a GTR + F + I + Γ4 substitution model, recommended by the “ModelFinder” model fitter included IQ-TREE. Branch robustness was assessed using the ultrafast bootstrap approximation with 1000 bootstrap replicates^39^. The resulting ML tree was visualized and edited in FigTree (https://github.com/rambaut/figtree/releases/tag/v1.4.4). TempEst v1.5 was used for investigating the temporal signal of our molecular phylogeny^40^. Analysis of the extreme values of residuals (tangential deviation from the regression line) permitted us to spot problematic sequences. Those genomes were discarded from the following steps. BEAST v1.10.4 was used for Bayesian Monte Carlo Markov chain (MCMC) analysis to estimate the divergence time of nodes^41^. We retained the simplest model: strict clock and constant population size with the HKY substitution model and four gamma categories. BEAST runs were completed checking chain convergence and sufficient sampling of the posterior space (ESS > 200). The final chronogram was generated using TreeAnnotator v1.10.4^41^.

### Ethical statement

The study was approved by the institutional committee of the Institute Pasteur of Bangui. For the experiments realised out of CAR, material transfer agreements were established with regards to national and international policies. All the experiments carried on mice were done in accordance with relevant guidelines and regulations on the use of laboratory animals including the ARRIVE guidelines.

## Results

### Bioinformatics analysis

De novo assembly of the raw reads from Illumina sequencing allowed us to obtain two sequences with lengths of 3390 nt and 7390 nt for the S and L fragments, respectively. Then, mapping back all the raw reads identified a total of 9,275,654 reads corresponding to the arenavirus. The mean depth was of 1902 × and 155 × for the S and L fragments, respectively. Mapping also made it possible to identify a total of 77,133 reads from MinION sequencing. After mapping MinION data on these two sequences, the mean coverage was 18 × and 3x. The taxonomic assignment using three different classification tools was performed only on the dataset that contained only reads corresponding to our virus. Taxonomic assignments showed that the percentage of reads from the AnRB-3214-Illumina dataset assigned as belonging to the *Arenaviridae* family ranged from 0.02 to 0.4% whereas for reads belonging to the AnRB-3214-MinION dataset, taxonomic assignment percentages were 0.01% and 0.88% for Centrifuge^32^ and Kaiju^31^, respectively (Table 1).

**Table 1:** Percentages of reads identified as an arenavirus using different classification tools. The highest number of reads classified as belonging to arenaviruses were obtained with Kaiju tool for both MinION and Illumina libraries.

	Number of reads	Kraken2 (%)	Kaiju (%)	Centrifuge (%)
**MinIon**	**50,010**	**0.12**	**0.88**	**0.01**
Dilution 10%	5001	0.16	1.04	0.03
Dilution 1%	500	0.26	1.04	0
Dilution 0.10%	50	0	0	0
**Illumina**	**9,275,654**	**0.07**	**0.45**	**0.02**
Dilution 10%	927,565	0.07	0.46	0.02
Dilution 1%	92,756	0.07	0.45	0.02
Dilution 0.10%	9276	0.05	0	0.02

**Figure 2:**
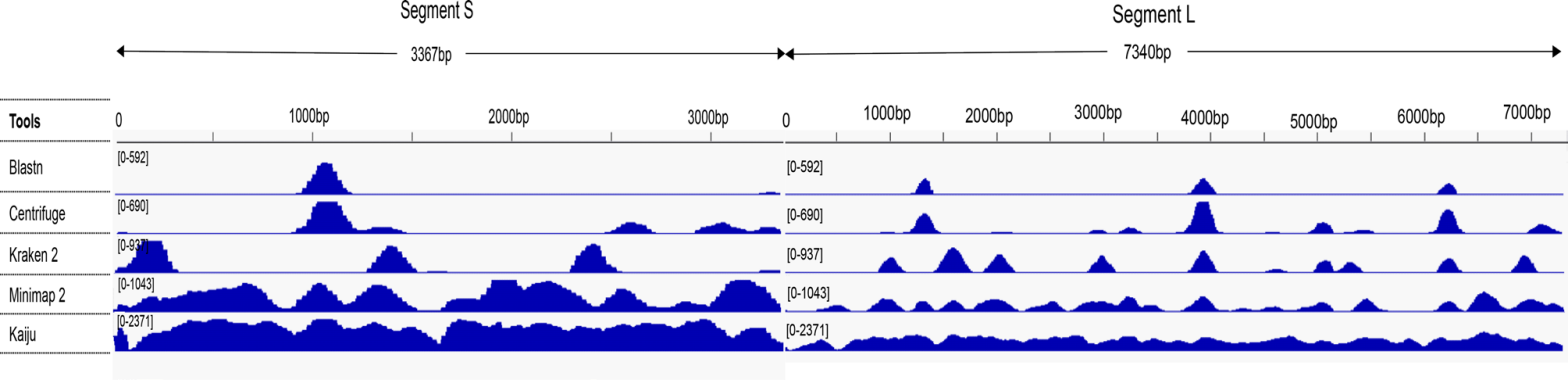
Map of the raw reads on the de novo assembled AnRB3214 genome. The figure shows the coverage and depth of arenavirus reads retrieved with different tools on the small (S) and large (L) segments of strain AnRB3214. Minimap2 and Kaiju afforded the most extensive coverage of both segments with a depth of more than × 1000. Blastn, Centrifuge and Kraken 2 map the reads only at certain genomic regions with low coverage of other portion of the genome.

Moreover, the percentage of reads from the computer-diluted Illumina datasets assigned as belonging to the *Arenaviridae* family only varied from 0.050 to 0.070% and from 0.020 to 0.024% for Kraken2^33^ and Centrifuge, respectively. The variation was also low with Kaiju (0.45 to 0.46%) except for the 0.1% subset for which no reads were assigned. Excluding the 0.1% subset in which no MinION reads could be classified, the percentages in the other datasets ranged from 0.03 to 1.04% for computer-diluted datasets from MinION sequencing (Table 1). In parallel to these three classification tools, two other tools were used to identify viral reads, a mapping tool (Minimap2^30^) and a homology search tool (BLASTN) using the sequence of the closest variant available in the GenBank (Mobala virus). From the AnRB-3214-Illumina dataset, a total of 26,726 reads (0.29%) were identified with Minimap2, but only 2364 (0.025%) were identified with BLASTN. Finally, although only 0.45% of the reads were classified with Kaiju, almost 100% of both the S and L segments were covered. The second best coverage was obtained with Minimap2 with 95% (573 positions uncovered), but using either BLASTN or the other two classification tools (Kraken2 and Centrifuge), the coverage percentages were lower, varying between 17% (BLASTN) and 45% (Kraken2) and were concentrated on only certain regions of the different fragments (Fig. 2).

### Genome analysis

We obtained two genomic segments, an S segment of 3390 nt and an L segment of 7391 nt, each segment encoding two non-overlapping open reading frames (ORFs) in an ambisense organization typical of arenaviruses. The S segment encodes GPC [position 146 to 1618] and NP [position 3190 to 1646] and the L segment encodes the Z protein [position 153 to 458] and the RdRp protein [position 7256 to 570].

Nucleotide sequences and the deduced amino acid sequence of AnRB3214 strain were compared with those of other OW arenaviruses species. The S and L segments of the AnRB3214 strain are phylogenetically very distant from the other described Ippy virus strain DakAnB188d^42,43^, with respectively 64% and 56% nucleic acid similarity, evidence that they belong to two distinct virus species. Although the two strains were identified in two locations in the CAR from *Arvicanthis* sp. (DakAnB188d) and *Praomys* sp. (AnRB3214) rodents almost 10 years apart, the AnRB3214 strain was classified as an Ippy virus due to cross-reactivity with DakAnB188d. Serum-based assays were widely used for the classification of viruses in the 1970-1980s and viruses were historically assigned to antigenic groups (Group A, Group B) given their antigenic relationships.

Additionally, for the S segment, at the nucleic acid level, the AnRB3214 strain displayed 79%, 72%, 70% and 68% similarity with Mobala virus, Gairo virus, Luna virus and Morogoro virus, respectively (Table 2). Similar trends were observed when comparing the L segment, where these viruses are the closest relatives with however divergences of 22%, 32%, 38% and 39%, respectively. Gairo, Luna and Morogoro viruses have been isolated from *Mastomys natalensis* in Tanzania and Zambia and the Mobala virus has been identified in Bouboui village in *Praomys* sp. from the CAR one year prior to the isolation of strain AnRB3214 in Botambi, another village of the same prefecture. When considering each protein in their respective segments, we observed an increase in the level of conservation. At the amino acid level, there was a high level of conservation for GPC with 94%, 83% and 82% of similarity with Mobala, Gairo, Luna, and Morogoro viruses (Table 2). The level of similarity with the other arenaviruses was low for the NP, Z and RdRp proteins. The Z protein of AnRB3214 was less conserved at the protein level than at the nucleic level and showed higher homology with that of Mobala virus, but less than 70% similarity with other close relatives, such as the Gairo and Luna viruses. Furthermore, the AnRB3214 NP appears to have been truncated of 43 to 78 amino acids (aa) at the 5′ end during the evolution of the virus. The AnRB3214 strain NP protein contains 515 aa, but OW arenavirus NP length ranges from 558 to 593 aa^44,45^. Indeed, when compared to NP of OW arenaviruses like Lassa, Mopeia, Mobala, Gairo and Luna, the NP deletion of AnRB3214 corresponds to the first 53 aa of these viruses. This feature was already observed at the nucleic acid level with the NP gene length of 1545 nt with the other arenavirus NP genes ranging from 1674 to 1779 nt.

**Table 2:** Similarities of strain AnRB3214 with other Old-World arenaviruses at the nucleic acid (NA) and amino acid (AA) level and accession number.

Virus	Accession No.		Date of isolation	Host	Country	Small (S) segment					Large (L) segment				
						Similarity (%)	GPC		NP		Similarity (%)	Z		RdRp	
	S segment	L segment					NA (%)	AA (%)	NA (%)	AA (%)		NA (%)	AA (%)	NA (%)	AA (%)
Mobala	AY342390.1	NC_007904.1	1980	*Praomys* sp*.*	CAR	79	83	94	74	83	78	82	83	80	87
Gairo	NC_026246.1	NC_026247.1	2012	*M. natalensis*	Tanzania	72	74	83	68	77	68	76	66	69	73
Luna	AB693148.1	AB693149.1	2010	*M. natalensis*	Zambia	70	73	83	65	69	62	76	68	64	65
Morogoro	NC_013057.1	NC_013058.1	2004	*M. natalensis*	Tanzania	68	71	82	66	70	61	72	63	63	63
Mopeia	DQ328874.1	DQ328875.1	1977	*M. natalensis*	Mozambique	68	71	82	65	70	61	75	64	63	64
Gbagroube	GU830848.1	nd	2005	*M. setulosus*	Ivory Coast	66	68	80	63	66	nd	nd	nd	nd	nd
Lassa	KM821904.1	KM821905.1	2013	*H. sapiens*	Sierra Leone	65	68	75	63	67	58	70	65	60	60
Wenzhou	KM386660.1	KM386661.1	2014	*R. norvegecus*	China	64	63	73	62	64	56	64	59	58	55
Ippy	DQ328877.1	DQ328878.1	1970	*Arvicanthis* sp*.*	CAR	64	66	75	61	65	56	66	65	58	56
Mariental	NC_027134.1	KP867641.1	2012	*M. namaquensis*	Namibia	63	66	77	60	64	52	63	64	53	49
Menekre	GU830862.1	nd	2005	*Hylomyscus* sp.	Ivory Coast	63	66	77	61	64	nd	nd	nd	nd	nd
Okahandja	NC_027135.1	NC_027137.1	2012	*M. namaquensis*	Namibia	62	63	69	59	61	56	69	57	56	53
Loie River	KC669698.1	KC669693.1	2008	*Bandicota* sp*.*	Thailand	63	64	73	56	59	56	67	64	58	55
Merino Walk	NC_023764.1	NC_023763.1	1985	*M. unisulcatus*	South Africa	61	63	71	59	60	57	65	55	59	55
LCMV	KJ603308.1	KJ603307.1	2014	*M. musculus*	USA	60	59	62	58	58	51	62	52	54	48
Lunk	NC_018710.1	NC_018711.1	2010	*M minutoides*	Zambia	57	58	61	57	57	50	64	53	50	45
Dandenong	EU136038.1	EU 136039.1	2008	*H. sapiens*	Australia	58	59	63	57	57	50	62	56	53	48
Lujo	NC_012776.1	NC_012777.1	2008	*H. sapiens*	Zambia	53	49	48	55	51	47	62	47	50	45
Souris	NC_039012.1	KP050226.1	2013	*Praomys* sp*.*	Cameroon	37		14	33	14	55		58	55	52

GPC: glycoprotein precursor; NP: nucleoprotein; Z, zinc-binding protein; RdRp: RNA-dependent RNA polymerase; CAR: Central African Republic; nd: no data, the L segment sequence is not available; *M. natalensis*: *Mastomys natalensis*; *M. setulosus: Mus setulosus*; *R. norvegecus: Rattus norvegecus*; *M. namaquensis*: *Micaelamys namaquensis*; *M. musculus*: *Mus musculus*; *M. minutoides*: *Mus minutoides*; *H. sapiens*: *Homo.*

**Figure 3:**
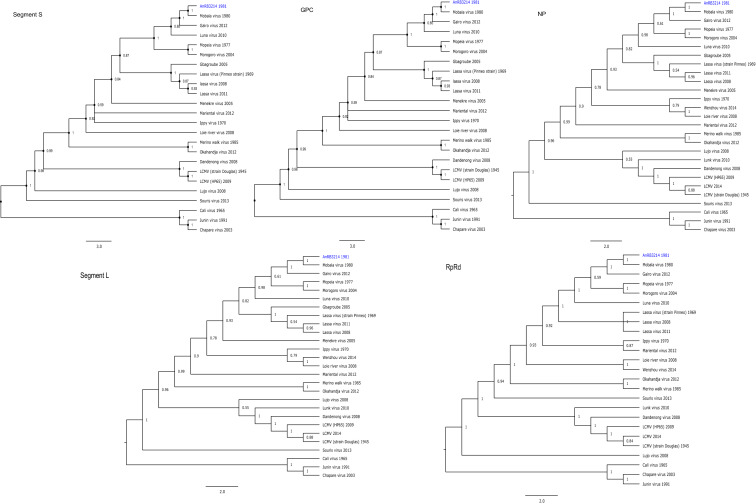
Phylogenetic trees built on the large (A) and small (B) segments. The phylogenetic trees were built using the whole genome segment under the maximum-likelihood method and the GTR-I-Γ4 substitution model with the 1000 bootstrap replicates. The trees show two groups of arenaviruses, one from the Old World (OW) and the other from the New World (NW). AnRB3214 (in blue) forms a cluster with Mobala, which is supported by a bootstrap value of 100. The phylogenetic trees were built in MEGA X v10.1.1 (https://www.megasoftware.net/).

Phylogenetic trees were built for each segment and coding region using references available in GenBank. All the tree topologies confirmed the conclusions drawn from the pairwise comparison of the sequences. For example, strain AnRB3214 belongs to the cluster comprising the Mopeia, Morogoro, Luna, Gairo and Mobala viruses (Fig. 3).

**Figure 4:**
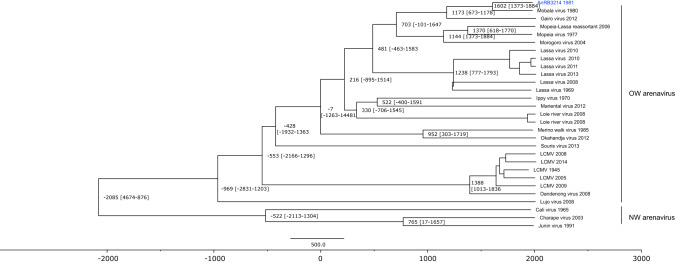
Molecular clock analysis of strain AnRB3214 using BEAST v1.10.4. The clock shows two monophyletic arenavirus groups which seems to have diverged some 5000 years ago. AnRB3214 strain (in blue) diverged from an ancestor in common with Mobala virus around year 1602 [year 1373- year 1884]. BEAST v1.10.4 (https://www.beast2.org/) was used for the Bayesian Monte Carlo Markov chain (MCMC) inference and the chronogram was generated with TreeAnnotator v1.10.4 (https://beast.community/treeannotator).

The AnRB3214 strain is genetically very divergent from the Ippy virus DakAnB188d strain, because they are found in two distant groups in the phylogenetic trees. Moreover, strain AnRB3214 shares a most recent common ancestor (MRCA) with Mobala virus. Molecular clock analysis shows that the two viruses diverged from one another around the year 1600 AD with a substitution rate of 4.28 × 10^–4^ subt/site/year (1374–1885, 95% highest posterior density (HPD)) (Fig. 4).

## Discussion

In this study, the AnRB3214 strain was sequenced on two sequencing platforms, Illumina and MinION, and we used a diverse set of taxonomic classification and mapping tools to retrieve arenavirus-specific reads from the obtained datasets. We obtained 77,133 and 9,275,654 reads specific to arenaviruses from the MinION and Illumina datasets, respectively. It is no surprise that we obtained less reads from MinION sequencing as this technology produces longer reads compared to Illumina. Additionally, the high amount of reads obtained during our experiment is representative of the type of sample that was used (here, cell culture supernatant) compared with direct sequencing of biological samples in which the viruses are present at low abundance with high levels of the host nucleic acid background^26,46^. When using reference viral genome to recover arenaviruses reads in our AnRB3213 dataset, the best read identifications were obtained with Kaiju and Minimap2. Few, if any, studies have compared tools for the classification of reads, either from long reads (MinION) or short reads (Illumina) for the analysis of divergent viruses. Minimap was initially developed for long reads, but has been updated to work with smaller paired-end reads, through a genuine management of gaps^30^. Furthermore, Minimap2 has been shown to be a good tool for the alignment of nanopore reads, with a higher speed than GraphMap although with comparable accuracy^47^. Contrary to Kraken2 and Centrifuge which primarily classify nucleotides, Kaiju uses amino acid sequences instead of genomic sequences for metagenomic classification. This feature allows Kaiju to overcome the limitations introduced by the redundancy of the genetic code and therefore make it suitable for investigating divergent organisms^31^. For more insight on our dataset and sequencing approaches, randomly generated subsets corresponding to 10%, 1%, and 0.1% of the overall initial outputs were generated for subsequent analysis to simulate a situation similar to that of a primary biological sample, i.e. from infected rodent reservoirs with low viral load. We showed that, except for the 0.1% dilution subset using Kaiju, arenavirus reads were detected by all the classification tools, although at varying rates. However, this test also demonstrated the limitations that can arise in the detection of such viruses in their reservoirs because the corresponding reads will be present in limited amounts.

OW mammarenaviruses are zoonotic viruses hosted by rodents of subfamily *Murinae*, which includes approximately 129 genera and 584 species. Members of about eight rodent genera are recognized as harbouring arenavirus species across the African continent^6,7^. For example, *Praomys* rodent species have a very extended geographic range and can be found across Sub-Saharan Africa. Ippy virus, Souris virus and Mobala virus have been isolated from *Praomys* rodents and *Praomys* clade rodents host almost all the OW arenaviruses^48^. Here, we described the AnRB3214 strain which was initially identified as an Ippy virus in 1981 in Botambi, CAR based on serological cross-reactivities. We demonstrated that strain AnRB3214 is distinct from the only other Ippy strain described to date, strain DakAnB188d, which also originates from the CAR. This classification was certainly due to cross-reactivity between the two viruses, because immunological assays were often used for virus identification and taxonomic assignment in the 1980s. Furthermore, “Ippy virus” has already been referred to as a complex comprising at least two viral species. Immunological microcompliment fixation data rapidly corroborated the presence of at least two distinct strains of Ippy virus, with low cross-reaction level, one of which was found in an *Arvicanthis* sp. rodent and the other in a *Praomys* sp. rodent^7^. With the description of the AnRB3214 strain, we confirm the huge arenavirus diversity in CAR rodents and the importance of the molecular characterization of viruses stored in biobanks.

Species delineation among arenaviruses is based on many criteria, including antigenic cross-reactivity, geographic distribution, host species and divergence at the amino acid level^49^. For OW arenaviruses, the interspecies amino acid cut-off divergences vary according to the protein: 8.6–20.4% (GPC), 10.2–21.5% (NP), 24.2–30.2% (Z) and 20.8–36.6% (L)^49^. Even so, some authors consider 12% divergence at the amino acid level in the NP enough to distinguish between two OW arenaviruses^50,51^. However, the latter criterion raises significant limitations, especially when considering the intra-species diversity of Lassa virus and NW arenaviruses^48,52^. Compared with Mobala virus at the amino acid level, strain AnRB3214 showed 6%, 13%, 17% and 17% genetic distance for the GPC, RdRp, Z and NP genes, respectively. Mobala virus and strain AnRB3214 were both isolated from unspecified *Praomys* rodents in the CAR in 1980 and 1981, respectively. Given the wide diversity of rodent species found in the forest areas of Central Africa, their morphological identification is often difficult and may sometimes require the use of molecular tools. Unfortunately, the absence of reference sequences for few rodent species mean that they cannot be identified^53^. Furthermore, *Praomys* rodents, which are subdivided into at least 20 putative species, are morphologically similar to *Mastomys* and *Hylomyscus* rodents^54,55^. Given that all these rodent species are abundant in CAR and that it is sometimes impossible to distinguish them on morphological criteria alone, it is difficult to confirm that strain AnRB3214 was isolated with certainty from a rodent of the *Praomys* genus. Mobala virus has also been isolated in Botambi, the same village from where strain AnRB3214 originates. Moreover, field and experimental evidence suggest that co-infections of a rodent species by two different arenaviruses are possible, although it has not yet been reported in the literature^6,54^. It is therefore difficult to conclude whether strain AnRB3214 is distinct from Mobala virus or not, especially because no further information is available for either virus. Molecular clock analysis under the strict clock model shows that the MRCA of strain AnRB3214 and Mobala virus can be dated back to year 1600 AD, almost 400 years ago. OW arenaviruses are thought to have originated nearly 3000–7000 years ago, as illustrated by LCMV speciation events which are estimated to have occurred around 1000 to 5000 years ago and the spread of Lassa virus from Nigeria to Mali and Ivory Coast some 1000–2000 years ago^2,55–57^. However, molecular clock analysis within Lassa virus lineages shows more contemporary virus speciation events, of less than 100 years up to 700 years^58,59^, similar to what was observed for strain AnRB3214. Additionally, the substitution rate deduced for strain AnRB3214 matches that of other arenaviruses, ranging from 3.3 × 10^–4^ to 6.3 × 10^–4^ subt/site/year, rates similar to those for Gbagroube virus, LCMV or Lassa virus^55,59,60^. Hence the circumstances that have led to the diversification of Mobala virus and strain AnRB3214 are probably similar to those observed within the Lassa virus lineages.

The AnRB3214 NP protein seems to be shorter than that of Mobala virus or the other OW arenaviruses, as it shows a ~ 53 aa deletion at the beginning of the N-terminal domain. Arenavirus NP proteins are essential for many aspects of the viral life cycle, such as the formation of the ribonucleoprotein complex when associated with RdRp, and the interference with the host immune system^45,61^. The N- and C-terminal domains have been shown to be the main NP regions directly involved in NP-NP oligomerisation, ribonucleoprotein complex formation and RNA synthesis. In comparison with the results obtained through an amino acid mutagenesis experiments in the N-terminus using the Tacaribe virus, a NW arenavirus^44,62^, we can conclude that the deletion observed in the AnRB3214 NP protein may not impact its biological function. However, further investigations are needed to understand the origin and consequences of this truncated nucleoprotein on viral particles.

More studies are needed to understand the implications of strain AnRB3214 for public health, especially in the CAR where civil unrest and political instability force the population to resort to feeding on forest-dwelling rodents. Although the prevalence of antibodies against Mobala virus in human population was associated with its prevalence in the rodent reservoirs^17,19^, cross reactivity with arenavirus strain AnRB3214 cannot be rule out.

## Conclusion

OW arenavirus diversity remains to be explored. Here, we revealed that strain AnRB3214, classified as an Ippy virus, is clearly a species close or identical to Mobala virus. This article shows once again the usefulness of molecular tools for taxonomic assignment, especially considering archived viral strains obtained using classical virology tools and classified based on antigenic similarities. In the CAR, 22 viral isolates identified as Ippy virus have been reported from *Lemniscomys striatus*, *Praomys* spp., *Arvicanthis* spp. and *Mastomys* spp. rodents, of which 16 were isolated from *Praomys* spp.^7,63^. As seen in our study, these strains can represent distinct arenaviruses, especially because a specific arenavirus is usually associated with a single host species. Therefore, this study opens new ways to characterize the remaining CAR arenavirus strains, and especially those classified as Ippy virus and improve knowledge on the ecology of OW arenaviruses.
